# Killing of *Gyrodactylus salaris* by heat and chemical disinfection

**DOI:** 10.1186/s13028-016-0202-y

**Published:** 2016-03-23

**Authors:** Perttu Koski, Pasi Anttila, Jussi Kuusela

**Affiliations:** 1 Production Animal and Wildlife Health Research Unit, Finnish Food Safety Authority Evira, Elektroniikkatie 3, 90590 Oulu, Finland; 2 Perämeren Kalatalousyhteisöjen Liitto, Piuhatie 8, 90620 Oulu, Finland; 3 Lahti University of Applied Sciences, Niemenkatu 73, 15140 Lahti, Finland

## Abstract

**Background:**

*Gyrodactylus salaris* is a monogenean, which has collapsed tens of wild Atlantic salmon populations. One of the means of preventing the spread of the parasite is the disinfection of the fishing equipment, which is used in the rivers having susceptible salmon populations. Little is known about the dosage of disinfectants against *G. salaris*. There are not standards for the testing of disinfectants against multicellular parasites. The present investigation developed a method to test disinfectants and examined the effectiveness of heated water and a commercially available disinfectant (Virkon S) in killing *G. salaris*. Individual *G. salaris* worms were followed under the microscope during treatment with heated water or Virkon S disinfectant blend. The logarithm of the time needed to kill the parasite was used as a dependent variable in linear regression. The upper 99.98 % prediction line for the dependent variable was used to obtain a value resembling the time needed for a 4 log reduction of the microbial pathogen, which is commonly used as a criterion for disinfectants. Also 6 log reduction was applied.

**Results:**

Exposure to a relatively low temperature was found to kill the parasite. Even 5–50 min treatment (=10–100 times the 99.98 % upper prediction value) with heated water at 40 °C might be used. This would enable the utilisation of hot tap water in the disinfection of fishing gear. The present practice of 1 % Virkon S for 15 min was also found to kill the parasite.

**Conclusions:**

The follow-up of single parasites of a test population and the use of the calculated upper predictive line in the regression analysis offers a method to analyse the effects of disinfectants on parasites like *G. salaris*. The results of our tests give possibilities for using disinfection methods, which may be more acceptable by the fishermen than the present ones.

## Background

*Gyrodactylus salaris* Malmberg 1957 was first identified on farmed Atlantic salmon (*Salmo salar* L.) at a fish farm in the Baltic Sea catchment area [1]. Since the 1970s, *G. salaris* has collapsed populations of wild Atlantic salmon in tens of Norwegian rivers and one Russian river [2, 3]. The parasite is widespred in the Baltic Sea catchment area [4–8]. Measures to prevent the spread of the parasite include prohibition of the transport of live fish to rivers containing wild Atlantic salmon unless the source of the fish is known to be free of *G. salaris* [9], barriers to stop fish migration upstream from infected river areas to uninfected ones, and eradication of infection in rivers by chemical treatment [3, 10, 11].

Although the risk of the spread of *G. salaris* by fishing equipment was not regarded very big [12], have national authorities in Finland, the United Kingdom and Norway provided guidelines or legal regulations on the disinfection of fishing equipment in order to prevent the spread of *G. salaris* to water systems free of the parasite [13–15]. Temperature (heating of equipment to 60 °C for 1 h) and the use of a commercial disinfectant blend (Virkon S) have been advised in addition to complete drying or freezing of the equipment. The suggested concentration–time combination for Virkon S, when mentioned in these instructions, has been at least 1 %–15 min. Some fishermen, especially anglers, fear that the use of disinfectants will harm their valuable equipment. This might reduce their willingness to undertake proper disinfection and perhaps jeopardise the preventive measures.

To our knowledge there are no international standards for the testing of the effect of disinfection of *G.* *salaris*. The European Committee for Standardisation has not provided norms for the testing of antiparasitic disinfectants [16]. The guidelines of the German Association of Veterinary Medicine provide advice for parasite eggs and Coccidia oocysts, but not for adult worms [17].

The present investigation examined the effectiveness of heated water and a commercially available disinfectant (Virkon S) in killing *G. salaris*. A simple testing method was developed to examine the lethal effect of disinfection on *G. salaris*. The aim was to examine the time needed to kill the parasite, when lower temperatures and concentrations of Virkon S than the present recommendations are used.

## Methods

### Fish

All rainbow trout (*Oncorhynchus mykiss* Walbaum) used in our experiments were one summer old, ca. 20–25 cm in length and obtained from a commercial fresh water fish farm in Northern Finland. The fish were transported to the laboratory in hatchery water and were maintained in two 500 l plastic fish tanks before the experiments. The tanks were sited in a thermostat-regulated room with a constant temperature of 10 °C. Before the tests involving increasing temperature, the fish and parasites were acclimated to 6 °C for 1 week. The fish were not fed during their stay in the laboratory. Tanks were aerated and had an internal recirculation of water with a sand filter.

### Parasites

Because it was assessed too difficult to get the worms unharmed onto the fishing equipment, *G. salaris* in their natural habitat, on the fish fin, were used. The rainbow trout were infected with rainbow trout type *G. salaris* (GenBank accession number AF479750) at the farm, from where they were transported to the laboratory. The species of *Gyrodactylus* was determined by molecular analysis of the mitochondrial CO1 sequence as described in [18].

The parasite survival was followed in the test system at 10 °C (before acclimatizing the fish and parasites to 6 °C). The parasites were found to live up to 85 h. After 48 h the fin and the parasite were already covered with a thick layer of slime and detached epithelium from the fin, but the parasites remained alive.

### Test design

In thermal treatments, each fish was killed with a blow on the head and fins were immediately cut while the fish were submerged in tank water. Parasitized fins were individually placed in a Petri dish in heated water from a tank water container. Parasites (1–3 individuals at a time) were continuously observed and the survival time was recorded with a stopwatch. When the parasite complex (including mother *G. salaris* and daughters in her uterus) stopped moving, the worm was gently irritated with an insect needle. In many cases, the parasites responded to irritation. The stopwatch was halted when the parasite did not move nor react to needle stimulation. The fins were handled similarly in the testing of Virkon S. Parasites were observed and the survival time was measured as in the tests with elevated temperature.

The possible recovery of *G. salaris* after the termination of its movements was tested by transferring the fin and attached parasite to fresh water. The amount of the disinfectants concurrently transferred on the surface of the parasite and fin was found to be negligible, as the median volume of water carried with the moved fin was ca. 37 µl (7 weight measurements). If this volume of 1 % Virkon S solution was mixed with the 65 ml of freshwater used in the test, the resulting concentration of Virkon S would be ca. 5.7 × 10^−4^ %. Based on the results it was concluded that the worms, which had lost their ability to move, could not recover and were dead.

### Tests with elevated temperature

**Fig. 1:**
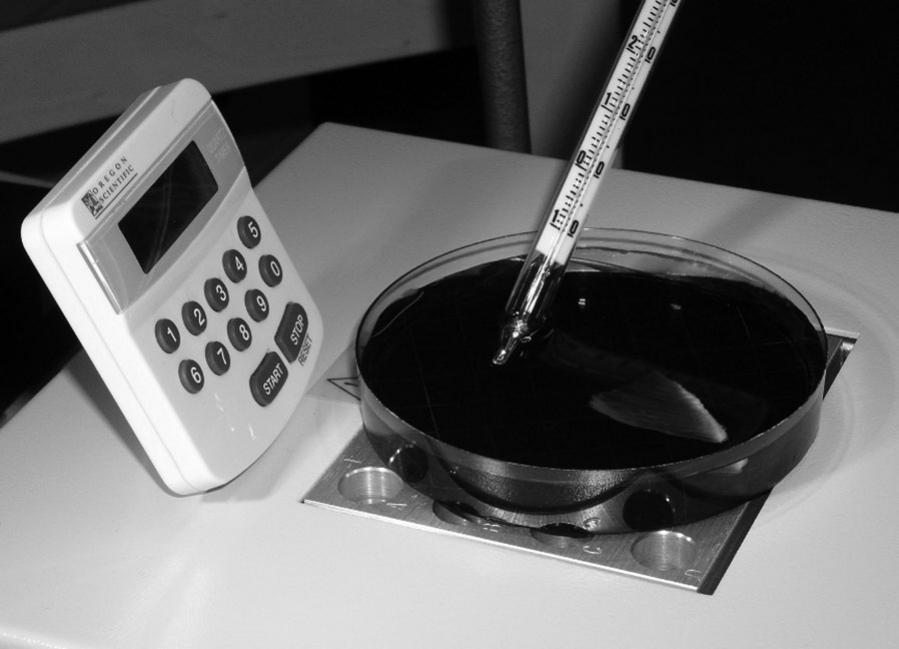
Test apparatus. *Gyrodactylus salaris* individuals were attached on a rainbow trout fin that was placed in 65 ml of fish tank water. The temperature of the water was maintained (or raised by heating with a desk lamp in the second experiment on heat disinfection) by the heat block under the Petri dish and followed with the thermometer. The survival time of individual parasites was recorded with the stopwatch

Thermal treatments were performed using a thermostat-regulated heating block (Fig. 1). In addition to the heating block, the desk lamp also heated the water. In the first test, 65 ml of tank water in a Petri dish was quickly heated to the test temperature (25, 30, 35 or 40 °C). The fin with the 1–3 parasites on it was then put to the preheated water and the time for the death of the parasite(s) recorded according to the protocol presented in the paragraph ‘Test design’. The water temperature was monitored during the tests, and the water was not aerated.

In the second test the temperature was slowly elevated from 6 °C until each worm ceased moving. This varied between 760 and 2359 s depending on the speed of the elevation of the water temperature. The rate of increase of the temperature was regulated by changing the distance of the desk lamp from the microscope. The time when the worm no longer moved even after the irritation by an insect needle was recorded as the time of death.

### Tests with Virkon S

Virkon S is a commercial oxidising disinfectant blend. The product used in the experiments described here (produced by Antec Int. Ltd, UK.) was labelled as containing the following: potassium peroxymonosulphate, sodium hexametaphosphate, sodium alcylbenzenesulphonate, malic acid, sulphamic acid, sodium chloride, fragrance and an indicator dye. Virkon S is widely used in the disinfection of livestock premises and in aquaculture.

All experiments with Virkon S solution were performed at 10 °C. Virkon S concentrations of 0.01, 0.05, 0.1 and 1.0 % were tested. The time until the mother and the daughter parasite did not move even after the irritation with an insect needle was recorded and used as the time of the death of the parasite.

### Statistics

Regression analysis was used to test the association between the temperature and survival time and between Virkon S concentrations and survival time. Logarithmic transformations of the original parameters were used, because there appeared to be difficulties in fulfilling the requirement of linearity in regression analysis [19]. The residuals of the logarithmic time variable followed a normal distribution.

The upper 99.98 % prediction line in the regression analysis was used as an estimate for the time needed to reduce the *G. salaris* population by 4 log. The 6 log reduction was counted on the basis of only 10^−6^ of the normally distributed population lying outside ±5× standard deviation of the mean.

Statistical analysis was carried out using the analytical software package SPSS for Windows version 22 [20].

## Results

### Effect of water temperature on the survival time of G. salaris

*Gyrodactylus salaris* was sensitive to treatment with warm water (Table 1), as the individual worms only survived for 6–18 s in 40 °C. The water temperature and the time until the cessation of movements were found to be associated and the following linear regression formula was obtained (R^2^ = 0.98, *P* < 0.001):

**Table 1:** The survival times of *Gyrodactylus salaris* in heated water and Virkon S

Temperature (°C)	+25	+30	+35	+40
Survival time [median (range)]	119.7 (67.4–209.3) min	12.4 (6.9–21.6) min	50.5 (36–98) s	9 (6–18) s
N	18	36	40	23
Virkon S (%)	0.01	0.05	0.1	1.0
Survival time [median (range)]	11.7 (3.4–34.2) min	3.1 (1.8–4.8) min	2.4 (1.4–4.5) min	14 (8–28) s
N	54	54	55	53

The survival times of single worms in different water temperatures (*upper*) and Virkon S (*below*)

*N* number of worms tested

$${\text{log TIME}} = 8. 7 2\,{ - }\,0. 20 \times {\text{TEMPERATURE }}\left( {^\circ {\text{C}}} \right).$$

**Fig. 2:**
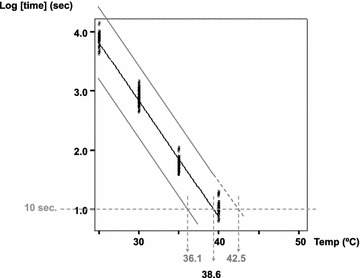
Relationship between the time to death of *Gyrodactylus salaris* and the water temperature. Scatter diagram, linear regression line (*middle line*) and the 99.98 % prediction lines for future individual observations (*upper* and *lower lines*) relating the fixed disinfection water temperatures (25, 30, 35 and 40 °C) to the logarithm of the survival time of *G. salaris*

On the basis of this and Fig. 2 it can be concluded that there is a clear killing effect of the increased temperature. Another regression was also tested in which both variables were logarithmically transformed, but R^2^ remained the same (0.98).

The higher 99.98 % individual prediction line indicates a survival time of ca. 30 s for *G. salaris* in 40 °C. If the 99.98 % confidence limits for the prediction interval are extrapolated to the temperature at which the predicted survival time of the parasite would be 10 s, 42.5 °C would be sufficient to stop all movement of the parasite. The 1-s treatment temperature would be 47.6 °C. The more critical requirement of 6 log reduction [7]—ca. 5 times the standard deviation from the mean—in 40 °C would be 34 s. This is not very much higher than the 4 log reduction time.

**Fig. 3:**
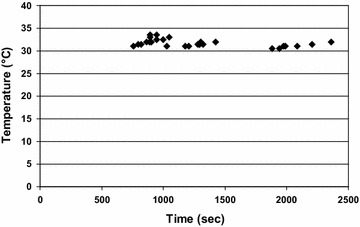
Lethal temperature of *Gyrodactylus salaris*. The temperature at which *G. salaris* were killed when the water temperature was slowly elevated from 6 °C to the lethal temperature. The time of the death of *G. salaris* is plotted on the x-axis

The results of the experiment in which the temperature was gradually raised are illustrated in Fig. 3. It appeared that *G. salaris* had a fairly constant lethal temperature of 30.5–33.5 °C, irrespective of the time taken (13 min 20 s–40 min) to reach this temperature. In the regression analysis the regression coefficient was 0.00 (R^2^ = 0.22).

### Survival time in Virkon S

The survival of *G. salaris* in Virkon S is shown in Table 1. The time until the cessation of movement of *G. salaris* and the Virkon S concentration were found to be associated and the following linear regression formula was obtained (R^2^ = 0.93, *P* < 0.001):

$${\text{log TIME}} = { - }0. 4 7{-}0. 8 4 {\text{ log CONCENTRATION}} .$$

**Fig. 4:**
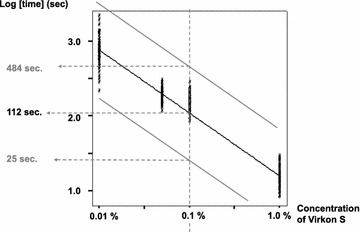
Relationship between the time to death of *Gyrodactylus salaris* and the concentration of Virkon S. Scatter diagram, linear regression line (*middle line*) and the 99.98 % prediction lines for future individual observations (*upper* and *lower* lines) relating the logarithm of the concentration of Virkon S (%) to the logarithm of the survival time of *G. salaris*

The test results are illustrated in Fig. 4. The higher 99.98 % individual prediction line indicates a survival time of 484 s for *G. salaris* in a 0.1 % solution (one tenth of the standard concentration) and 17 s in 1 % solution of Virkon S. The more critical requirement of 6 log reduction [21] in 1 % solution was counted to be ca. 102 s.

## Discussion

Freshly cut rainbow trout fin is most likely a better living environment for *G. salaris* than the surface of fishing equipment. The parasites may tolerate the action of disinfectants, if they thrive well. This may divert the estimations of the survival time of the parasite in a safe direction. On the other hand the presence of extraneous substance like slime may be more probable in the surrounding of the parasite on the fin than on the surface of fishing equipment. The influence of such extraneous material probably divert the estimations to other direction [22], when the applicability of our results to the practical disinfection of fishing gear is considered.

Disinfection that reduces a bacterial, fungal or viral titre by 4–5 logs is commonly regarded as effective [16, 23, 24]. The 6 log reduction is used, when medical devices are designated to be sterile according to [21]. The 99.98 % prediction interval for individual *G. salaris* was used here for counting the 4 log reduction of *G. salaris*. One ten thousandth part of the *G. salaris* population used in the test would stand longer disinfection times than the upper 99.98 % prediction line. Killing times above this line were considered to reduce the *G. salaris* population by 4 log. The basis for selecting the 4 log reduction instead of 5 log was the assumption that relatively low number of *G. salaris* do attach to fishing gear under real conditions.

### Effect of water temperature on the survival of G. salaris

Study of the biology of *G. salaris* has naturally focused on physiological water temperatures [25]. According to Office International des Epizooties [26], the tolerance of *G. salaris* to temperatures above 25 °C is unknown. The temperature and time needed to kill *G. salaris* in this study was much lower than that used for the general disinfection of fish farms [26]. Heat can be used in the disinfection of fishing equipment, but the advice of the [13]−60 °C for 1 h—is very much higher than the time needed to kill the parasite with heated water in this study. The short time of survival (movement) of the parasite at high temperature did not allow temperatures higher than 40 °C to be tested. The extrapolated temperature (42.5 °C, Fig. 2) required to kill the parasite after a 10-s treatment is, however, close to the highest tested temperature and probably a good estimate of the actual lethal temperature in dipping disinfection lasting 10 s, if 4 log reduction would be the goal.

A margin of safety may be applied in the practical disinfection procedures in addition to the 4–5 log reduction of the pathogen. In the review by [27] of the general protocols for effective iodophor disinfection of viral haemorrhagic septicaemia (VHS) virus on salmonid eggs, this margin appeared to increase the treatment time by a factor of ca. 10. The present norm for heat treatment (60 °C for 1 h) could probably be much lowered without jeopardising the efficiency of disinfection. A treatment time as low as 5–50 min (=10–100 times the 99.98 % upper prediction value) with heated water at 40 °C could be used, if a factor of 10–100 was applied.

The results of the second test examining the effect of temperature on *G. salaris* survival supported the use of heated water: the parasite died when the increasing water temperature reached a fairly moderate level.

The killing effect of the temperature treatments might be associated with the decreased oxygen concentrations of the heated water and not the temperature per se. There is ca. 8.1 mg l^−1^ dissolved oxygen (D.O.) in fully saturated water at 25 °C and 6.6 mg l^−1^ at 40 °C. Because the heated water was only kept for a short time in a broad-mouthed vessel (Petri dish), it is unlikely that the saturation would have significantly decreased from the full saturation in the tank water of live fish. We are unaware of the limits for *G. salaris,* but the host fishes do not thrive at D.O. <5 mg l^−1^ [28]. It is unlikely that the requirements of *G. salaris* exceed those of the host fish.

In this study the parasites were, however, treated in warm water, whereas practical disinfection procedures usually involve moist or dry heat, the thermal conductivity of which is much lower. The heated water in this study could therefore be more effective in killing the parasite than disinfection with moist or dry heat. The total energy required to elevate the temperature of the parasite is small due to the small size of the animal.

### Effect of low concentrations of Virkon S on the survival of *G. salaris*

Virkon S proved to be efficient in killing *G. salaris*. Even one tenth of the standard concentration of the solution resulted in death of the parasite in less than 10 min. This would enable the use of Virkon S in lower concentrations than currently recommended. If a similar margin of safety as discussed in the section on heat disinfection (factor 10–100) was applied, the disinfection time for 0.1 % Virkon S would, however, be impractical (1 h 37 min–16 h 8 min).

The present guideline of 1 % Virkon S for 15 min is long enough both on the basis of the evaluation according to 4 log reduction with a safety factor of 10–100 or 6 log reduction.

## Conclusions

The demonstrated vulnerability of *G. salaris* to even a moderate heating of the water has practical applications. The lethal temperature to *G. salaris* is unlikely to damage even the most delicate angling equipment such as the line and flies. The short time needed to kill the parasite at temperatures over 40 °C allows the use of routine facilities such as hot tap water to disinfect small fishing equipment. The use of moist or dry heat in sauna or drying cabinet would need more evaluation before the present advice could be safely lowered.
